# Microsaccades Track Location-Based Object Rehearsal in Visual Working Memory

**DOI:** 10.1523/ENEURO.0276-23.2023

**Published:** 2024-01-19

**Authors:** Eelke de Vries, Freek van Ede

**Affiliations:** Department of Experimental and Applied Psychology, Institute for Brain and Behavior Amsterdam, Vrije Universiteit Amsterdam, Amsterdam 1081 HV, The Netherlands

**Keywords:** attention, eye movements, microsaccades, oculomotor system, task-dependent, visual working memory

## Abstract

Besides controlling eye movements, the brain's oculomotor system has been implicated in the control of covert spatial attention and the rehearsal of spatial information in working memory. We investigated whether the oculomotor system also contributes to rehearsing visual objects in working memory when object location is never asked about. To address this, we tracked the incidental use of locations for mnemonic rehearsal via directional biases in microsaccades while participants maintained two visual objects (colored oriented gratings) in working memory. By varying the stimulus configuration (horizontal, diagonal, and vertical) at encoding, we could quantify whether microsaccades were more aligned with the configurational axis of the memory contents, as opposed to the orthogonal axis. Experiment 1 revealed that microsaccades continued to be biased along the axis of the memory content several seconds into the working memory delay. In Experiment 2, we confirmed that this directional microsaccade bias was specific to memory demands, ruling out lingering effects from passive and attentive encoding of the same visual objects in the same configurations. Thus, by studying microsaccade directions, we uncover oculomotor-driven rehearsal of visual objects in working memory through their associated locations.

## Significance Statement

How humans rehearse information in working memory is a foundational question in psychology and neuroscience. To provide insight into the cognitive and neural bases of working memory rehearsal, we turned to microsaccades—small eye movements produced by the brain's oculomotor system. We reveal how microsaccades track memorized objects’ locations during memory rehearsal, even when object location is never asked about. This brings three advances. From a psychological standpoint, it demonstrates how rehearsal engages object locations. From a neuroscience standpoint, it demonstrates how such location-based rehearsal recruits brain circuitry that also controls our eyes. From a practical standpoint, it demonstrates how microsaccades are a relevant variable in cognitive neuroscience studies and can themselves be utilized to track working memory rehearsal across space and time.

## Introduction

The brain's oculomotor system, which controls our eye movements, has also been linked to the regulation of covert cognitive processes. To date, the cognitive involvement of the oculomotor system has been established most clearly in the studies of covert visual–spatial attention and the rehearsal of spatial information in working memory (Rizzolatti et al., 1987; Corbetta et al., 1998; Nobre et al., 2000; Hafed and Clark, 2002; Engbert and Kliegl, 2003; Moore et al., 2003; Theeuwes et al., 2009; Krauzlis et al., 2013; Hafed et al., 2015; Jonikaitis and Moore, 2019). In the current study, we tested whether the oculomotor system also contributes to the location-based rehearsal of multiple visual objects in working memory when object locations are never explicitly asked about. To address this, we studied microsaccades as a direct time-resolved output from the oculomotor system.

Studies in which participants are explicitly tasked with memorizing the locations of visual stimuli have revealed that the oculomotor system plays a role in the mnemonic rehearsal of these locations. Most directly, spatial rehearsal of visual locations has been shown to trigger eye movements to these locations (Olsen et al., 2014; Wynn et al., 2018). Complementary evidence comes from studies showing better visual–spatial memory when eye movements are allowed throughout the delay interval (Postle et al., 2006; Williams et al., 2013; Pearson et al., 2014; Souza et al., 2020; McAteer et al., 2023; but see also Godijn and Theeuwes, 2012; Loaiza and Souza, 2022), and worse memory when eye movements are prompted to other locations (Lawrence et al., 2004; Postle et al., 2006; Tremblay et al., 2006; Ohl and Rolfs, 2020). Neuroscience studies in humans (Courtney et al., 1998; Postle et al., 2000; Curtis et al., 2004; Jerde et al., 2012) and nonhuman primates (Armstrong et al., 2009; Merrikhi et al., 2017) have further revealed how spatial locations in working memory are represented in the frontal eye fields—a key cortical node in the brain's oculomotor system (Moore and Armstrong, 2003; Marshall et al., 2015). These studies collectively point to an important role for the oculomotor system in the rehearsal of spatial location information in working memory.

Besides the retention of locations, working memory enables us to retain object-specific visual features, such as object color and shape. The aforementioned oculomotor involvement in visual working memory may be specific to the rehearsal of locations (Postle et al., 2006; as suggested in McAteer et al., 2023). At the same time, we know from ample prior studies that space remains a profound organizing principle for object retention in working memory, even when the location of memoranda is never asked about. For example, spatial locations may help to keep memory objects separate and facilitate the binding of features belonging to the same memory object (Treisman and Zhang, 2006; Hollingworth, 2007; Abrahamse et al., 2014; Pertzov and Husain, 2014; Manohar et al., 2017; Schneegans and Bays, 2017). In addition, spatial locations may act as a medium for object selection and prioritization from working memory (Griffin and Nobre, 2003; Kuo et al., 2009; Theeuwes et al., 2011; van Ede and Nobre, 2023) and may serve “spontaneous” object rehearsal in working memory (Foster et al., 2017). It has remained unclear, however, whether location-based rehearsal of visual object features other than location relies on the brain's oculomotor system.

In a series of recent studies that focused on mnemonic selection (van Ede et al., 2019, 2020, 2021; Liu et al., 2022; de Vries et al., 2023), we have established how the brain's oculomotor system—as read-out through microsaccadic biases in gaze—participates in the focusing of specific visual objects held within the spatial layout of working memory, even when object location is never explicitly asked about. Building on this complementary work that focused on the phase of mnemonic selection and prioritization, in the current study, we focused on microsaccades during the working memory delay phase, to examine oculomotor contributions to the location-based rehearsal of multiple visual objects in working memory.

**Figure 1.**
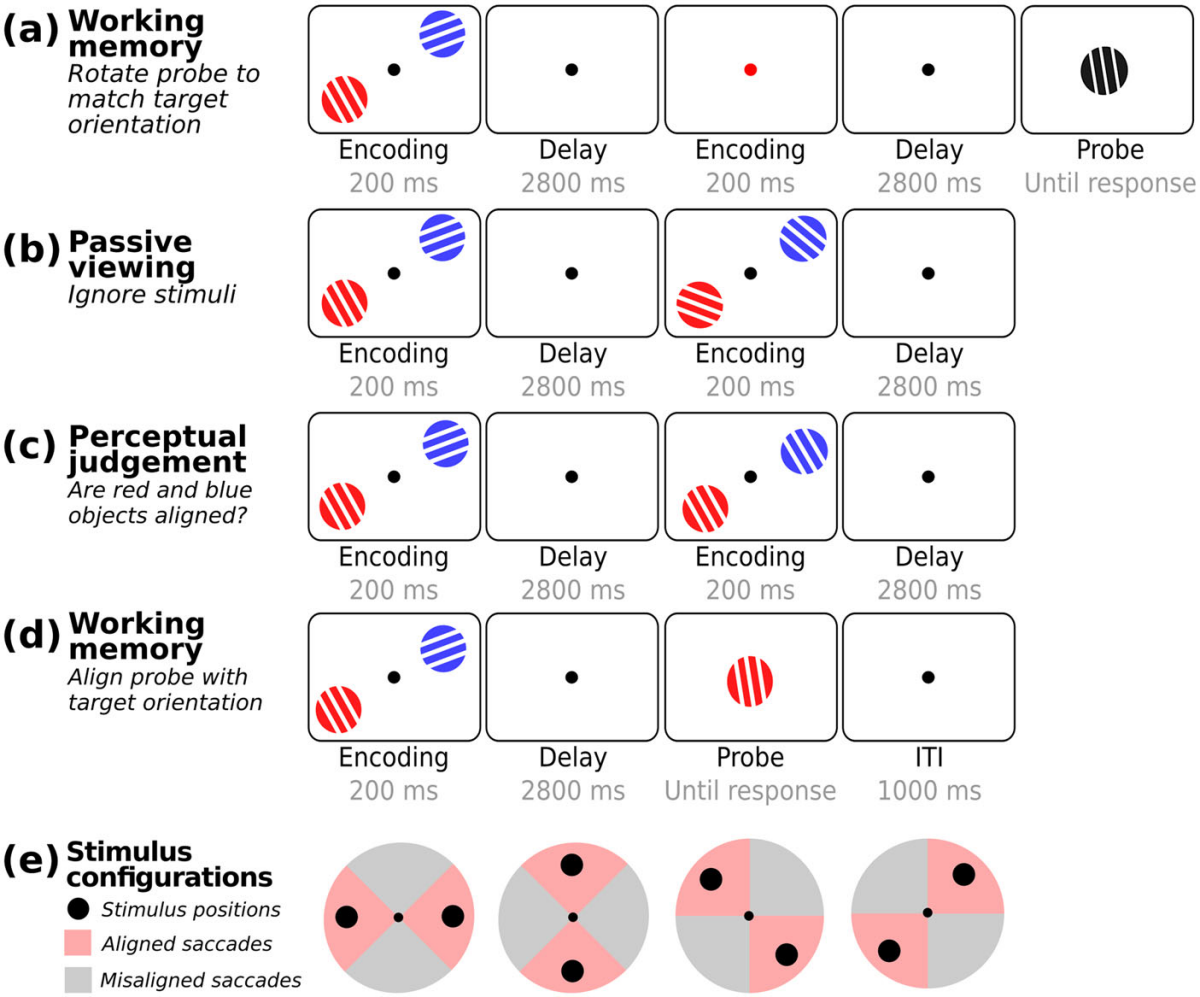
Task designs for Experiment 1 (***a–c***) and Experiment 2 (***d***) and criteria for saccade classification based on stimulus configurations (***e***). In every case, trials started with the bilateral presentation of two sample gratings, followed by a delay interval. ***a***, In Experiment 1, participants indicated whether a probe was oriented clockwise or counterclockwise with respect to the target memory object that was indicated by a color retrocue presented during the delay. ***b***, In the passive viewing condition of Experiment 2, participants were instructed to ignore the stimuli, while maintaining central fixation. ***c***, In the perceptual judgment condition of Experiment 2, participants had to indicate whether the orientations of the two simultaneously presented gratings in a given display were identical. ***d***, The task in the memory condition of Experiment 2 was the same as in the first experiment, only now the cue was integrated into the probe, by virtue of the probe's color. ***e***, Spatial arrangement of the four stimulus configurations during encoding (horizontal, vertical, and two diagonal). In Experiment 1, the two visual objects could occupy one of four configurations at encoding. In Experiment 2, the two visual objects always occupied either of the two diagonal configurations at encoding. Saccades were labeled as “aligned” when their direction was closer to the configurational axis of the visual objects during encoding (red areas) and “misaligned” when their direction was closer to the orthogonal axis (gray areas).

The current study aimed to address whether the oculomotor system contributes to working memory through location-based rehearsal of multiple visual objects, even when object location is never asked about. To address this, we capitalized on directional biases in microsaccades (Hafed and Clark, 2002; cf. Engbert and Kliegl, 2003; Liu et al., 2022) as a direct and time-resolved read-out of oculomotor engagement during working memory retention of two visual objects. By presenting memory objects in different spatial configurations at encoding (Fig. 1*e*), we were able to isolate and track object rehearsal—as reflected in microsaccades—through the incidental locations of the memoranda. Furthermore, by comparing directional microsaccade biases across different task demands with the same spatial configurations (Fig. 1*b–d*), we could disentangle the contribution of working memory rehearsal from the passive and attentive encoding of the same visual objects in the same configurations. Doing so, we reveal oculomotor rehearsal of visual objects in working memory through their associated—but in our case always task-irrelevant—locations.

## Materials and Methods

### Participants

We conducted two experiments with 25 healthy human volunteers in each (Experiment 1, mean age = 21.96 years, age range = 18–28 years, 13 women, 1 left-handed; Experiment 2, mean age = 21.76 years, age range = 18–37 years, 17 women, 1 left-handed). A sample size of 25 per experiment was determined a priori based on the use of the same sample size in relevant prior studies that used the same outcome measure to target complementary questions (van Ede et al., 2019, 2020, 2021; Liu et al., 2022; de Vries et al., 2023). The recruitment of participants for the two experiments was conducted independently. Data from all participants were included in the analysis. All participants received course credit or monetary compensation (€10 per hour), provided written informed consent prior to participation, and reported normal or corrected-to-normal vision.

### Task and procedure Experiment 1

**Figure 2.**
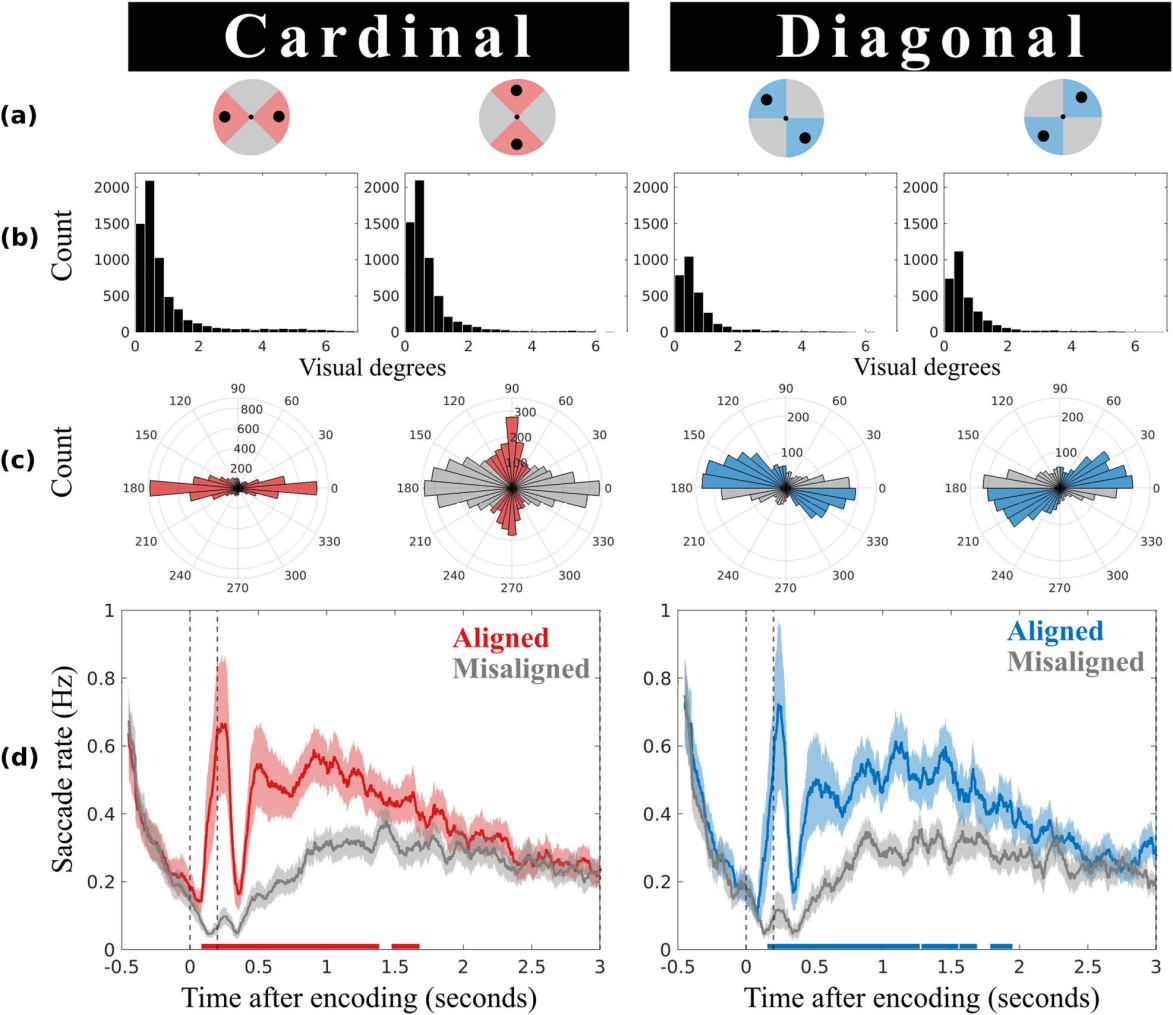
A directional microsaccade bias during working memory maintenance reveals location-based object rehearsal. ***a***, Spatial layout of the four stimulus configurations that were used in Experiment 1, with two solid black circles marking the locations of each stimulus pair at encoding. Saccades were classified as “aligned” when they were more closely aligned with the configurational axis of the visual objects (colored areas) and “misaligned” when they were more closely aligned with the orthogonal axis (gray areas). ***b***, Saccade size distributions of delay period saccades reveal that eye movements did not revisit the encoding locations of the memoranda. ***c***, Polar distributions of delay period saccades, with the colored bins corresponding to saccades along the aligned axis and the gray bins corresponding to saccades along the misaligned axis. ***d***, The time courses of the saccade rates (in Hz) for saccades along the aligned and misaligned axes. The horizontal lines at the bottom highlight statistically significant clusters. The shaded lines represent ±1 standard error of the mean. The two vertical dotted lines mark stimulus onset and stimulus offset.

Experiment 1 used a within-subject design in which we manipulated stimulus configuration at encoding. Participants performed a visual working memory task (Fig. 1*a*), in which they memorized the orientation and color of two objects presented on opposite points around a central fixation dot. The key manipulation was the variation of the stimulus configuration at encoding (horizontal, vertical, or either of two diagonal configurations; Figs. 1*e* and 2*a*). This manipulation enabled us to investigate whether microsaccades during the subsequent delay interval were more closely aligned with the configurational axes of the memory content, as opposed to the orthogonal axes—even if object location was incidental to the task because a location was never asked about.

Each trial started with a 1 s intertrial interval that featured a small fixation dot, followed by the simultaneous presentation of two memory objects for 200 ms and a delay interval of 2,800 ms. The color of the fixation dot then changed for 1,000 ms serving as a retrocue to instruct (with 100% validity) which visual memory object would be probed after the second delay interval (1,000 ms). The probe was always tilted at an angle of ±20 degrees relative to the cued memory target. The probe was always black and remained on the center of the screen until a response was provided. Participants had to match the orientation of the probe with that of the cued memory object by pressing the F key (counterclockwise) or the J key (clockwise).

The experimental stimuli were generated and presented on an LCD monitor with a solid gray background (ASUS ROG Strix XG248; 1,920 × 1,080 pixels; 240 Hz refresh rate) using Psychtoolbox (Brainard, 1997) for MATLAB. Participants were seated in a dimly lit room with their heads resting on a chin rest at approximately 70 cm from the display. The central fixation dot was black and had approximately 0.3° visual angle. The memoranda had a diameter of 1.6° and were presented with a horizontal and vertical displacement of 6.4°. The orientation of each stimulus was chosen randomly. The configuration (horizontal, vertical, and diagonal), color (red and blue; 21/165/234; 234/74/21), and target position of the memoranda were varied pseudo-randomly to ensure that all these features were distributed equally within each block. The duration of each experimental session, which had 12 blocks of 36 trials each (totaling 432 trials), was roughly 70 min. Participants prepared for the experiment by practicing 36 trials.

### Task and procedure Experiment 2

**Figure 3.**
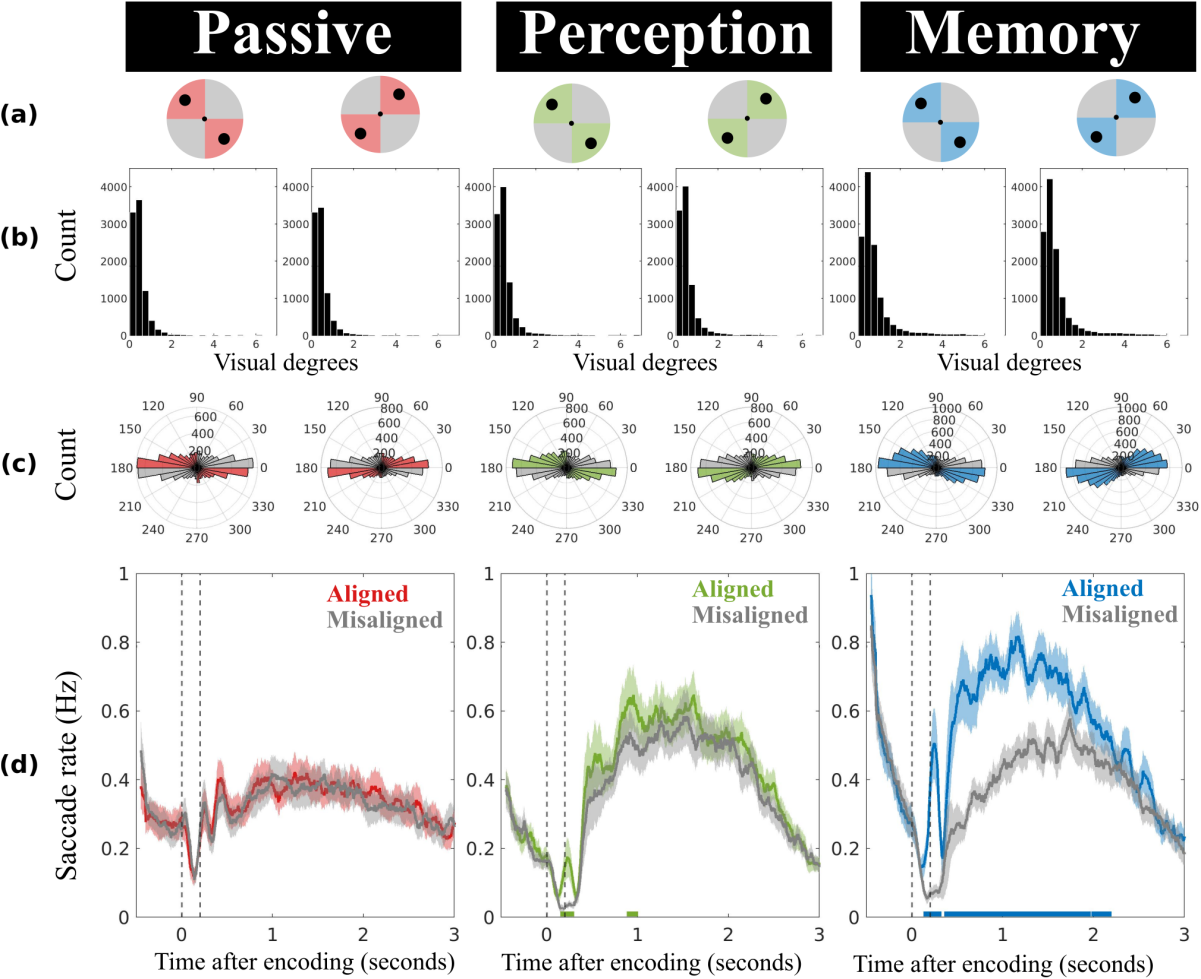
Directional bias in microsaccades is driven by working memory demands. ***a***, Spatial layout of the stimulus configurations that were used in Experiment 2, with the locations of each stimulus pair indicated by the two solid black circles. Saccades were classified as “aligned” when they were more closely aligned with the configurational axis of the visual objects (colored areas) and as “misaligned” when they were more closely aligned with the orthogonal axis (gray areas). ***b***, Saccade size distributions of delay period saccades. ***c***, Polar distributions of delay period saccades, with colored bins representing saccades along the aligned axis and gray bins representing saccades along the misaligned axis. ***d***, The time courses of the saccade rates (in Hz) for saccades along the aligned and misaligned axes. The horizontal lines at the bottom highlight clusters that are statistically significant. The shaded lines represent ±1 standard error of the mean. The two vertical dashed lines denote the onset and offset of the stimulus.

Experiment 2 was designed to determine whether the directional microsaccade bias observed in Experiment 1 reflected the active location-based rehearsal of memory content or instead reflected a lingering trace from encoding. To this end, Experiment 2 followed the same overall setup as Experiment 1 but introduced different task demands—specifically passive viewing and perceptual judgment blocks—in addition to working memory blocks similar to those in Experiment 1. These control conditions were included to distinguish the effects of active working memory rehearsal from passive and attentive encoding of identical visual objects. Moreover, to reduce factors, in Experiment 2, we always presented objects in either of the two diagonal encoding configurations (Fig. 3*a*).

In the passive viewing blocks (Fig. 1*b*), participants were instructed to ignore the stimuli, while maintaining central fixation. In the perceptual judgment blocks (Fig. 1*c*), participants had to indicate whether the orientations of the two objects that were concurrently presented in any given display were identical (∼7% of encoding displays in perceptual judgment blocks were such target trials) by pressing the spacebar within 1 s after stimulus onset. The task in the working memory blocks (Fig. 1*d*) was the same as Experiment 1, only now the color cue (informing the target memory object) was integrated into the probe by making the probe itself match the color of either memory object. The orientation of each stimulus was chosen randomly, but unlike in Experiment 1, there was always at least a 20° difference in orientation between the two objects in a trial (except for target trials in perceptual judgment blocks, which had identical orientations). We kept the 20° separation consistent across the three tasks to reduce uncertainty and ensure that in the perception blocks, participants could reliably distinguish between target trials (objects with identical orientations) and nontarget trials (objects with nonidentical orientations). Participants completed 24 blocks, each with 48 trials (totaling 1,152 trials). The order of the blocks was randomized in sets of three (i.e., passive, perception, memory). Each block began with a brief overview of the instructions for the upcoming task. Participants practiced 32 trials of each of the three task types in preparation for the experiment.

### Data acquisition and preprocessing

The right eye position was continuously recorded with an EyeLink 1000 Plus (SR Research) at a sampling rate of 1,000 Hz. The eye-tracking data were converted into ASCII format and analyzed with MATLAB and FieldTrip. Eye blinks were detected with an algorithm based on fluctuations in the pupil data (Hershman et al., 2018) and linearly interpolated. The data were then segmented into epochs from −500 ms to 3,000 ms relative to encoding onset. We estimated participants’ attentiveness to the memory task by measuring reaction times and excluding trials with responses slower than the mean reaction time +4 standard deviations (following an iterative procedure until no more outliers remained). We also did not analyze trials from perception blocks that contained targets (identical orientations) or were misidentified as such (false detection). Following these criteria, only a small percentage of trials were eliminated in each experiment (Experiment 1: *M *= 1.51%, *SD* = 0.81%; Experiment 2: *M *= 3.64%, *SD *= 0.79%).

### Eye-tracking analysis

To detect gaze shifts (saccades), we build on an existing velocity-based detection approach that has been successfully employed and validated in prior studies (Liu et al., 2022, 2023a; de Vries et al., 2023). Saccade detection was achieved by calculating the Euclidean distance between successive gaze points in the horizontal and vertical planes, smoothing the resulting velocity vector (using the built-in “smoothdata” function in MATLAB, with a 7 ms sliding window), and identifying the onset of a saccade as the instant that the velocity exceeds a predetermined threshold. A trial-based threshold of five times the median velocity was employed to determine saccade onset, and a minimum interval of 100 ms was used between saccades to prevent multiple detections of the same saccade. We additionally refer the reader to Supplementary Figure 1 in Liu et al. (2022) that provides a detailed visual representation of example saccades, including velocity traces, thresholds, and the analysis pipeline for detecting saccades. Note how we here applied this to saccades in the two-dimensional plane considering both horizontal and vertical components of gaze and thus considering two-dimensional velocity profiles.

To index the incidental use of locations for working memory, we tracked the direction (angle) of saccades and classified them as “aligned” when their direction was closer to the configurational axis of the visual objects during encoding and “misaligned” when their direction was closer to the orthogonal axis. Specifically, saccades were considered “aligned” when their direction was within ±45° from either of the two memory items. Conversely, “misaligned” saccades were those within a ±45° range from the axis orthogonal to the two memory items. Figure 1*e* depicts these classification criteria visually for each of the four stimulus configurations. By adopting this precise classification approach, we ensured that detected saccades were either classified as aligned or as misaligned, but never as both. The resulting aligned and misaligned saccade rate time courses (expressed in Hz) were then smoothed (using the built-in function “smoothdata” in MATLAB) using a moving average with a 100 ms sliding window. Crucially, directions classified as aligned in one configuration (e.g., vertical stimulus arrangement) would be classified as misaligned in the complementary configuration (e.g., horizontal stimulus arrangement). Accordingly, by collapsing across configurations, we were able to isolate the effect of stimulus configuration by subtracting out any background preferred saccade directions that would be common across all configurations.

### Statistical analyses

To assess whether the oculomotor system is involved in the rehearsal of visual objects in working memory, we compared the time courses of aligned and misaligned saccade rates. This was statistically evaluated using a cluster-based permutation approach (Maris and Oostenveld, 2007), which effectively avoids the issue of multiple comparisons by evaluating the full dataspace under a single permutation distribution of the largest cluster. It groups neighboring time points that show significant differences between the two time courses into a cluster and then randomly permutes the condition labels to create a null distribution of the largest cluster that would be observed by chance. If the observed cluster size is larger than what would be observed by chance, the two time courses differ statistically. We used the FieldTrip toolbox (Oostenveld et al., 2011) to identify significant clusters using the default configuration settings (10,000 permutations; α level of 0.025 per side).

In Experiment 2, we additionally compared the directional microsaccade biases from our working memory condition of interest to each of the control conditions (passive, perception) using paired sample *t* tests. For this comparison, we extracted the effect of interest as the difference in aligned versus misaligned saccades rates, averaged across the full delay interval, as determined a priori.

### Code accessibility

Raw data and analysis code have been made publicly available via the Open Science Framework and can be accessed at https://osf.io/ud6g2/. The analyses were conducted in MATLAB on a Linux-based computing system.

## Results

### Experiment 1: microsaccades reveal location-based oculomotor rehearsal in visual working memory

Before turning to our main eye-tracking results, we ascertained that participants were able to perform the working memory task: participants had an average accuracy of 75.97% (*SD *= 11.37%) and an average reaction time of 1,019 ms (*SD *= 346 ms).

The main purpose of Experiment 1 (Fig. 1*a*) was to investigate whether the oculomotor system is involved in the maintenance of visual objects in working memory, even when object location is never asked about. The incidental use of location for working memory was tracked by classifying saccades as “aligned” when their direction was closer to the configurational axis of the visual objects at encoding and “misaligned” when their direction was closer to the orthogonal axis (as illustrated in Figs. 1*e* and 2*a*).

Figure 2*b* depicts the distribution of saccade sizes across the full 2.8 s delay period for trials with cardinal and diagonal stimulus configurations. The majority of saccades in the delay period were <1° visual angle (i.e., microsaccades; Hafed and Clark, 2002; Martinez-Conde et al., 2004; Engbert, 2006; Rolfs, 2009; Nyström et al., 2016; Poletti and Rucci, 2016) and did not revisit the initially encoded locations of the memory objects (which were presented at a distance of 6.4°).

Our main result concerns the direction of the identified saccades as a function of memory object configurations at encoding. Figure 2*c* depicts the polar distribution of all saccades detected during the delay period. The colored bins correspond to saccades along the aligned axis, while the gray bins correspond to saccades along the misaligned axis. Taking all of the saccades during the memory delay into account reveals how saccade directions were generally biased along the axis of the visual objects at encoding (i.e., the aligned axis). While there is generally a horizontal bias in cardinal configurations (Fig. 2*c*, left panels; consistent with Engbert and Kliegl, 2003; Hermens and Walker, 2010), we found a configural memory bias on top of this general preference for horizontal saccades. This memory configuration–specific bias is especially evident when comparing the two diagonal configurations, where the data is less distorted by the background horizontal bias (Fig. 2*c*, right panels).

To quantify this bias and track its evolution over time, we collapsed across horizontal/vertical and across the two diagonal configurations and counted saccades along aligned versus misaligned axes (relative to encoding configurations) as a function of time. Crucially, directions that were classified as aligned in one configuration would be classified as misaligned in the complementary configuration. Accordingly, by collapsing configurations, we could isolate condition-specific saccadic biases by subtracting out any “background” saccade direction preferences that would be common to all configurations. Further, note how the probe was always presented centrally, so any directional microsaccade bias could not reflect probe anticipation.

Figure 2*d* shows the saccade rates across time for the aligned (colored) and misaligned (gray) axes. A cluster-based permutation comparison of the time courses confirmed significantly higher saccade rates on the aligned than on the misaligned axis (indicated by the horizontal lines in Fig. 2*d*, cluster *p*'s = <0.001, 0.012 for the cardinal-configuration data; cluster *p*'s = <0.001, 0.003, 0.023, and 0.013 for the diagonal-configuration data). Importantly, this was not only evident during encoding but lasted for a considerable period of time well into the working memory delay interval.

Thus, these data show that during working memory maintenance of visual objects, saccades—particularly microsaccades—occur more frequently along the spatial axis where memoranda were incidentally encoded than along the orthogonal axis (where no stimuli were encoded). This is consistent with object rehearsal by the oculomotor system through location, even when object location is never asked about.

### Experiment 2: the microsaccade bias during the delay reflects working memory demands

In Experiment 1, microsaccades continued to be biased along the aligned axis well into a working memory delay. However, merely demonstrating that microsaccades remained biased in their direction during the delay does not necessarily imply that they reflect active memory maintenance, as such biases could also reflect residual, lingering consequences from prior sensory encoding.

Experiment 2 (Fig. 1*b–d*) sought to replicate the microsaccade bias during the memory interval (this time using only the two diagonal conditions; Fig. 3*a*) and to compare it to two control conditions in which participants either (1) passively viewed the same objects or (2) were required to make a perceptual judgment on the two objects (detect instances where both simultaneously presented objects had identical orientations) without subsequent working memory demands. If the bias truly reflects active working memory maintenance (rehearsal) processes, it should only be profound in our memory task, and not after passive presentation, or perceptual evaluation of the same objects, that we presented in the same configurations, followed by the same delay.

Before turning to our main eye-tracking results, we first confirmed that participants comprehended the working memory and the perceptual judgment tasks. In working memory blocks, the average accuracy was 70.57% (*SD *= 13.32%), and the average reaction time was 1,348 ms (*SD *= 616 ms). In perceptual judgement blocks, participants showed high hit rates following target displays (*M *= 83.38%, *SD *= 17.53%) and low false-alarm rates following nontarget displays (*M *= 2.60%, *SD *= 2.62%).

Having established that participants were able to do both tasks, we turn to our main eye-tracking results. Figure 3*b* shows the distribution of saccade magnitudes for passive, perceptual, and memory trials. As in Experiment 1, the vast majority of saccades occurred within the microsaccade range and again this was the case for all conditions. Figure 3*c* depicts the polar distribution of saccades during the delay period, again showing a clear bias on memory trials (in blue, on the right), but little evidence of a similar bias for the two control conditions with passive object presentation (in red, left) or the perceptual judgment task (in green, middle).

To quantify the bias and track its temporal profile, we again counted saccades along the aligned and misaligned axes, as defined by the incidental stimulus configurations at encoding. Figure 3*d* shows that saccades in passive blocks are equally likely to occur along the aligned and misaligned axes (cluster *p *> 0.2), indicating that stimulus presentation alone cannot account for the reported directional saccade bias in working memory trials. In perception blocks, we discovered a slight bias for saccades to occur more along the aligned axis (cluster *p*'s = 0.008 and 0.024), but this bias was relatively weak and appeared to persist for only about 1 s after stimulus onset, which was also the time limit for participants to indicate that the two objects were identical.

In comparison, and replicating the results of Experiment 1, the memory condition again reveals a profound directional bias that persisted well into the working memory delay period (cluster *p*'s = <0.001, 0.0034, 0.023, and 0.013). A direct comparison of the memory condition with both control conditions confirmed that the memory condition had a significantly stronger bias in microsaccade direction: the saccade rate effect (aligned minus misaligned saccades rates, averaged across the full a priori defined delay period) was significantly larger in memory blocks (*M *= 0.18, *SD *= 0.11) than that in passive blocks (*M *= 0.01, *SD *= 0.02), *t*_(24)_ = 7.87, *p *< 0.001, and perception blocks (*M *= 0.05, *SD *= 0.06), *t*_(24)_ = 6.31, *p *< 0.001.

These findings rule out that the observed saccade bias during working memory maintenance is due to lingering processing of the passive viewing or attentive encoding of salient visual objects per se. Instead, the prolonged directional saccade bias in the memory task must be attributed to working memory demands, which is consistent with an oculomotor-driven rehearsal of memorized visual object features (color and orientation) through their memorized locations.

### Microsaccade biases across both rehearsal and selection phases

We also explored how oculomotor behavior relates to different phases of the working memory task. This was intended primarily to help bridge our current work on the rehearsal of multiple memory contents to our prior work on the post-retention phase of focusing on a single memory item for guiding behavior. Importantly, while in our current work, we adopted a measure in which we compared aligned and misaligned saccades, in our prior work looking at mnemonic selection, we used a different measure that was optimized for tracking attentional focusing to the target memory content: the comparison between “toward” (toward the target) and “away” (away from the target but toward the nontarget) saccades.

**Figure 4.**
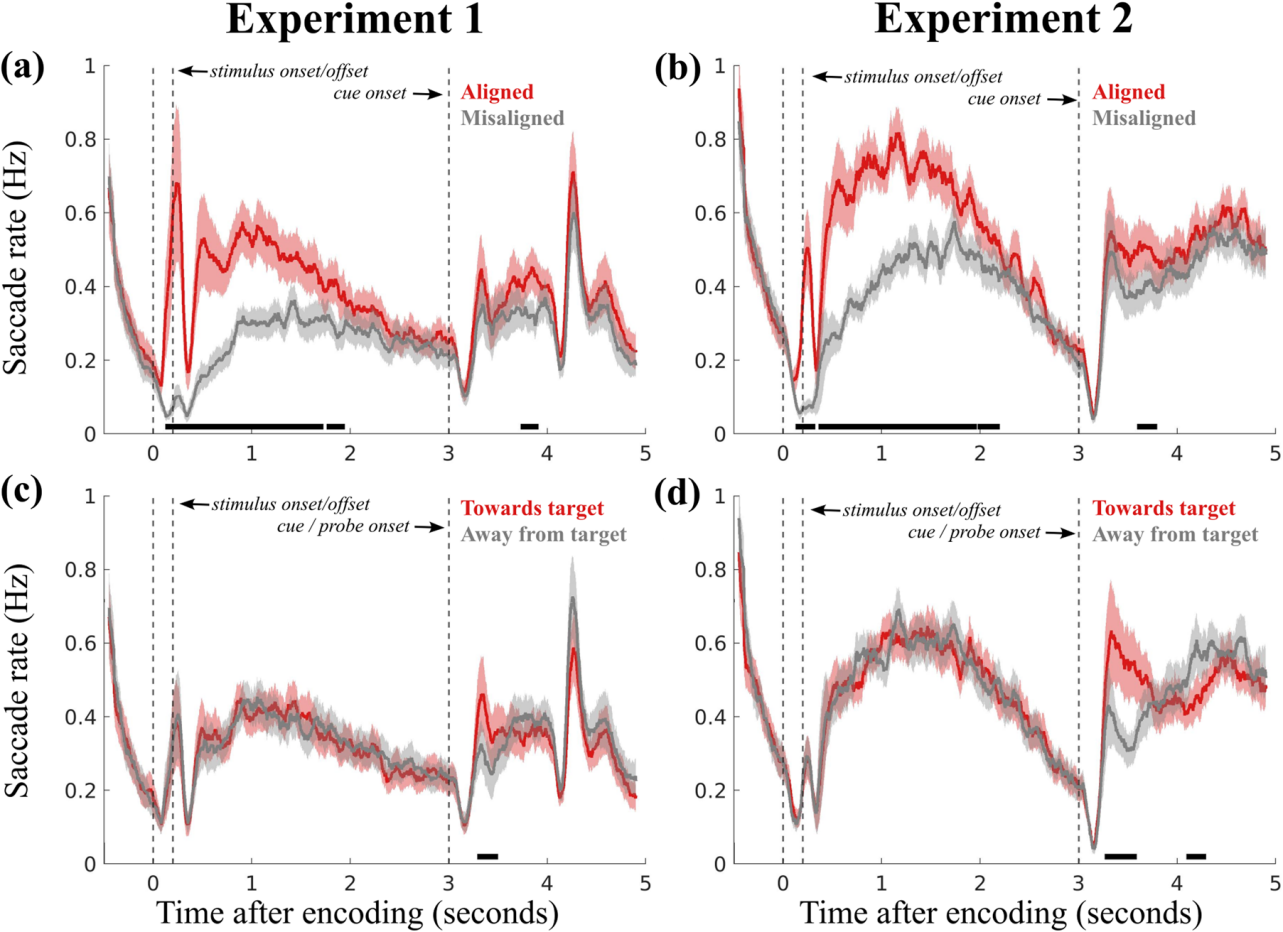
Comparison of oculomotor behavior across rehearsal and selection phases in visual working memory tasks. The aligned/misaligned classification is determined by the angular direction of saccades, within a range of ±45 degrees from the angle of both stimuli (Fig. 1*e*). The toward/away classification is binary and relates to the angular direction of saccades with respect to the cued target, within a range of ±90°. Panel (***a***) depicts average saccade rates categorized as “aligned” or “misaligned” in Experiment 1, extending the analysis to a 5 s window (compare Fig. 2*d*). Panel (***b***) illustrates aligned/misaligned saccade rates from Experiment 2, also within a 5 s window (compare Fig. 3*d*, right panel). Panel (***c***) shows average saccade rates categorized as “toward” or “away” from the cued target in Experiment 1. Panel (***d***) presents similar data for Experiment 2. The error bars denote ±1 standard error of the mean (SEM). The black horizontal lines at the bottom of each panel highlight clusters where the difference between aligned/misaligned and toward/away saccade rates is statistically significant.

For completeness, we show both comparisons in Figure 4, with time ranges including both rehearsal and selection phases. While our aligned and misaligned comparison did also capture the previously described selection bias in the post-cue (Experiment 1) and post-probe (Experiment 2) periods (Experiment 1, cluster *p*'s = <0.001, 0.015, 0.015, 0.025; Experiment 2, cluster *p*'s = 0.007, <0.001, 0.014, 0.01, 0.01), this bias appeared more clearly when relying on the toward versus away comparison (Experiment 1, cluster *p* < 0.001; Experiment 2, cluster *p*'s = <0.001, 0.002) that we had also adopted previously in our studies looking at mnemonic selection (van Ede et al., 2019, 2020, 2021; Liu et al., 2022; de Vries et al., 2023). Note how the latter comparison between toward and away was optimized for—and exclusively sensitive to—the period during mnemonic selection, given that “toward” and “away” (unlike aligned and misaligned) only become meaningful when it becomes clear which of the two memory items is the target.

### The relationship between the delay period microsaccade bias and subsequent behavioral performance

**Figure 5.**
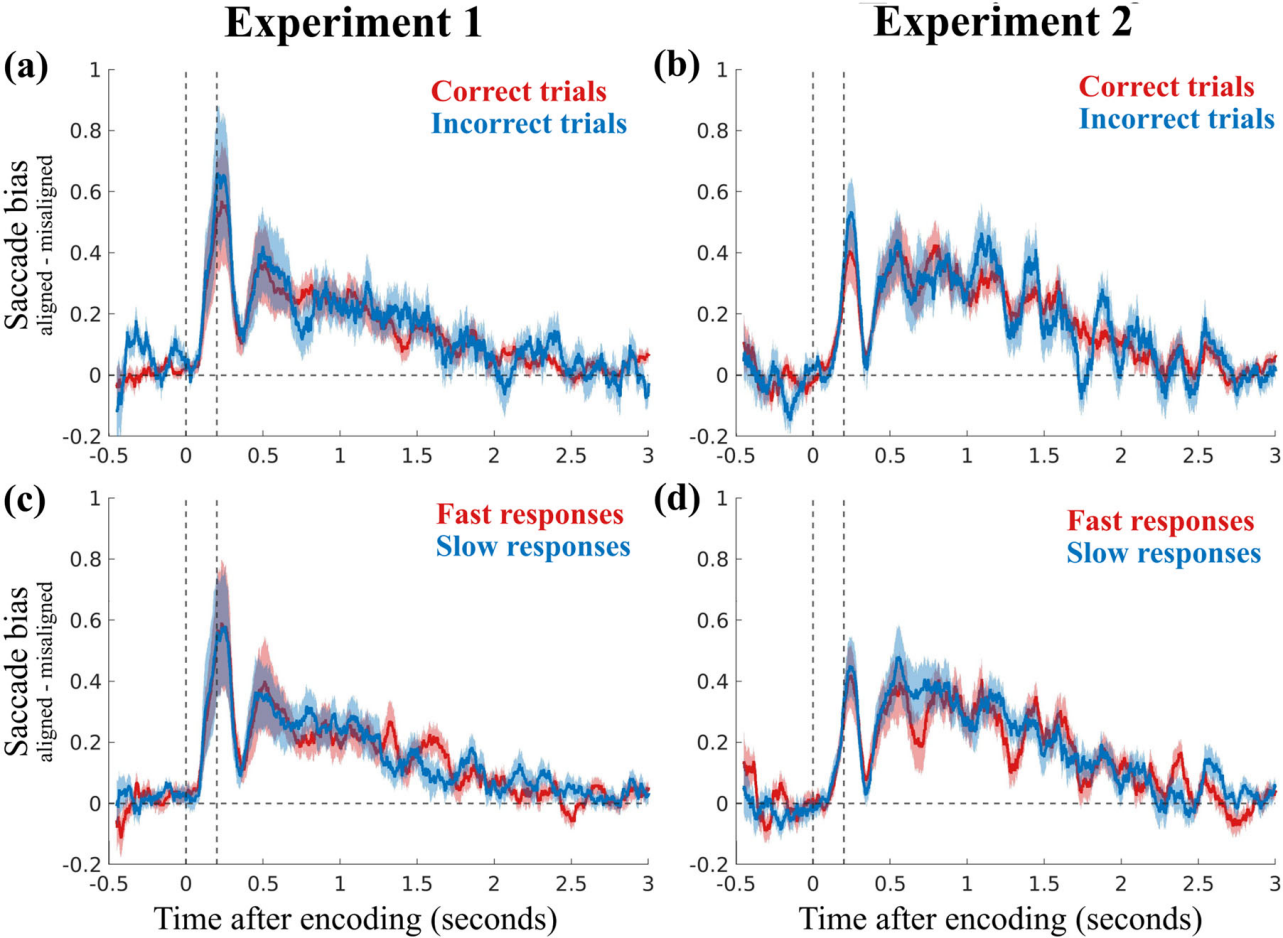
Examining the relationship between microsaccade bias and behavioral performance across experiments. The top two panels compare microsaccade bias (the difference between aligned and misaligned saccade rates) in Experiment 1 (***a***) and Experiment 2 (***b***) for trials with correct (red) and incorrect (blue) responses. The bottom two panels show the same microsaccade bias but for trials with answers that were faster (red) or slower (blue) than the median reaction time in Experiment 1 (***c***) and Experiment 2 (***d***). The two vertical dotted lines mark stimulus onset and stimulus offset. The shaded areas denote ±1 SEM. Cluster-based permutation tests did not reveal significant differences in saccade bias with respect to either performance accuracy or reaction time.

Even though our task was not optimized to assess the behavioral consequences of oculomotor rehearsal—given that the task involved retaining only two memory objects and did not require a precision report—we nevertheless conducted additional analyses to explore potential links between the observed microsaccade bias during working memory rehearsal and subsequent task performance. For this analysis, data from the working memory conditions of both experiments were partitioned based on response accuracy (correct vs incorrect responses) and reaction time (fast vs slow responses, based on a median split). We then considered the saccade bias as the difference between aligned and misaligned saccade rates and compared trials with high and low performance (Fig. 5). Cluster-based permutation tests did not reveal any significant difference in the observed saccade bias between trials with high and low performance.

## Discussion

It has been established that the brain's oculomotor system not only controls where we look but also participates in the rehearsal of spatial location information in working memory (Awh et al., 2006; Theeuwes et al., 2009; Olsen et al., 2014; Wynn et al., 2018; as reviewed in Jonikaitis and Moore, 2019; Souza et al., 2020). It has been called into question, however, whether this role extends to the visual working memory of objects whose location is never asked about (Postle et al., 2006; McAteer et al., 2023). By analyzing microsaccades as a direct and time-resolved output from the oculomotor system, we provide unique evidence that the brain's oculomotor system also participates in the rehearsal of visual objects in working memory, even when object location is never asked about.

Experiment 1 revealed how microsaccade directions remained biased during the working memory delay along the axis at which objects were incidentally presented at encoding. Experiment 2 replicated this central finding and ruled out lingering effects from passive or attentive encoding. By demonstrating directional biases in microsaccades during working memory maintenance, we thus reveal rehearsal of memorized visual object features through their incidentally encoded memorized locations and implicate the oculomotor system in such rehearsal. In the following, we consider and discuss three central questions raised by these findings.

### What may the observed microsaccade bias during working memory maintenance reflect?

Spatial biases in microsaccades have previously been linked to covert shifts of attention to peripheral locations (Hafed and Clark, 2002; Engbert and Kliegl, 2003; Yuval-Greenberg et al., 2014; but also see Horowitz et al., 2007; Willett and Mayo, 2023), including when directing attention within the spatial layout of visual working memory (van Ede et al., 2019; Liu et al., 2022; de Vries et al., 2023). Accordingly, our findings of directional microsaccade biases during working memory maintenance may reflect active attentional rehearsal or refreshing of memory representations (Oberauer, 2019). Though the location was never asked about, it may still facilitate rehearsal. This is consistent with prior research demonstrating that space remains important for organizing working memory content, even when location is never asked about (Kuo et al., 2009; Theeuwes et al., 2011; van Ede et al., 2019; Liu et al., 2022; de Vries et al., 2023). Our data go beyond this existing body of work by implicating the brain's oculomotor system in such incidental use of location and by demonstrating this role during working-memory maintenance, extending prior work implicating the oculomotor system in mnemonic selection (e.g., van Ede et al., 2019; Liu et al., 2022; de Vries et al., 2023).

By focusing on object rehearsal through location, our findings are also distinct from complementary recent studies that have linked directional biases in microsaccades to the rehearsal of visual shape and orientation information (Dotson et al., 2018; Mostert et al., 2018; Quax et al., 2019; Thielen et al., 2019; Linde-Domingo and Spitzer, 2023). In contrast to these studies where microsaccades directly tracked the target-relevant memory feature (shape or orientation), we used microsaccades to track incidental object locations that were never queried for report.

Consistent with the role of the oculomotor system in object rehearsal, complementary dual-task experiments have revealed enhanced memory for objects whose incidental locations became saccade targets during the working memory delay (Hanning et al., 2016; Ohl and Rolfs, 2020). This suggests some type of “refreshing” of memory objects when they become the target of a saccade. Our microsaccade observations were made without asking participants to make any eye movements and persisted for several seconds after encoding. Accordingly, our microsaccade observations may signal serial refreshing by the oculomotor system that may serve as a mechanism supporting object maintenance. This putative role in serial rehearsal is consistent with previous research on oscillating patterns in the directionality of microsaccades (Tian et al., 2016, 2018). In future studies, it will be interesting to address whether and how these microsaccade findings relate to recent complementary findings of serial object sampling in working memory (Peters et al., 2021; Pomper and Ansorge, 2021; Chota et al., 2022). While our data visualizations hint at potential rhythms in the working memory rehearsal bias, further studies and additional advanced analyses will be required to delineate and quantify the temporal dynamics of the spatial bias in microsaccade directions that we unveiled here.

It is also important to consider that the oculomotor system may perform more than one task. In our experiment, we asked participants to keep fixation on a central fixation dot. On top of this fixation instruction, we observed robust biases in fixational microsaccades in the direction of the configurational axis of the visual working memory contents. This suggests that the oculomotor system can serve dual functions: controlling eye position on the central fixation marker (Guerrasio et al., 2010; Hafed, 2011; Tian et al., 2016), while also participating in working memory rehearsal. Because of these dual demands, at least some of the saccadic bias we observed may result from re-fixations after an initial break of fixation in service of working memory (e.g., a refixation saccade to the left-bottom following a rehearsal saccade to the right-top memory item). How these dual functions operate in parallel in the oculomotor system (e.g., whether and how they are kept separate or interact with each other) remains interesting questions for future research.

### Why does the observed microsaccade maintenance bias diminish with time?

Experiment 2 demonstrated that the directional microsaccade bias was specific to memory demands, ruling out lingering effects from passive and attentive encoding. At the same time, despite ongoing memory demands, the bias diminished with time in the delay interval, and this was the case in both experiments. Interestingly, however, this appears not to be unique to the working memory signature that we uncovered here. For example, the contralateral delay activity (an EEG signature frequently implicated in working memory maintenance; Vogel and Machizawa, 2004; McCollough et al., 2007; Luria et al., 2016) similarly decays during the memory delay (Hakim et al., 2020; Jin et al., 2020; Roy and Faubert, 2023).

Even so, the observed decay raises the intriguing question of why this may occur and what it may reflect. One possible explanation is that rehearsal demands become less urgent as time passes. Early after encoding, active rehearsal may facilitate consolidation, while later in the delay active rehearsal may be less critical, and passive maintenance may suffice. For example, with time, working memory representations may become increasingly reliant on activity-silent mechanisms (through temporary changes in synaptic connectivity, see Mongillo et al., 2008; Stokes, 2015), rather than states of active rehearsal. Another possibility is that over time, location information simply becomes less relevant for working memory maintenance, as objects are gradually recoded to a less visual format, or shifted from peripheral to more foveal (Chambers et al., 2013; Fan et al., 2016) or global (Ester et al., 2009) cortical representations. Finally, it is conceivable that the anticipation of the probe stimulus itself may have affected our ability to measure robust microsaccade biases since stimulus expectation is known to reduce overall microsaccade rates (Olmos-Solis et al., 2017; Amit et al., 2019; Denison et al., 2019; Tal-Perry and Yuval-Greenberg, 2021). It is worth noting that these explanations are not mutually exclusive, as multiple (as well as additional) factors may contribute jointly; providing a relevant avenue for future research.

### Limitations and considerations in linking microsaccade bias to behavioral performance

Our study provides evidence for the role of microsaccades in tracking location-based object rehearsal in visual working memory. At the same time, additional analyses did not confirm a straightforward relationship between microsaccade directionality during the delay period and subsequent task performance metrics such as accuracy and reaction time.

One important consideration when interpreting this is that our task design was not optimized for assessing behavioral relevance. Our working memory task may not have been sufficiently demanding to reveal a clear benefit of oculomotor rehearsal, as it required the retention of only two memory objects (thus being relatively easy). Moreover, for simplicity, we used a two-alternative forced-choice (2-AFC) task, instead of asking participants to make a continuous recall report which may provide more sensitive data when correlating rehearsal signals to performance. In addition, even when participants responded incorrectly, this should not be interpreted as a lack of rehearsal; factors such as response mapping confusion may have also led to erroneous reports, even if rehearsal was successful. Likewise, correct responses do not necessarily indicate effective rehearsal, as the 2-AFC task permits a 50% chance of guessing correctly.

Second, performance is likely to be influenced by a variety of factors, including rehearsal (other factors that may influence performance include the quality of encoding, the degree of competition between memory objects, adequate memory and implementation of the appropriate response mapping, time on task, and so on). Furthermore, even when considering only “rehearsal,” successful rehearsal is likely to engage additional processes in addition to the process of revisiting memory objects’ encoded locations, as tracked here in microsaccades (i.e., the process of revisiting locations may be part of rehearsal, but it is unlikely to be the only process that determines rehearsal success). Given these considerations, it is thus unlikely to observe a one-to-one mapping between microsaccade rehearsal dynamics and memory performance.

A final point to consider is that rehearsal may not always be the strongest in trials with the best performance. It may be particularly relevant in trials with poor encoding, with post-encoding rehearsal serving as a compensatory mechanism to improve consolidation (which may be less required in trials with better encoding). It is thus conceivable that there may be opposing processes that could cancel each other out, making the relationship between microsaccade directionality and behavioral outcomes less straightforward.

Despite these challenges, our findings remain relevant for the broader understanding of working memory rehearsal, and they call for future studies with task designs optimized for discerning these behavioral consequences, perhaps incorporating higher demands on rehearsal (e.g., higher working memory load) and tasks with more informative response variability (e.g., continuous recall tasks). Indeed, in our prior work on gaze biases during mnemonic selection, we have observed behavioral relevance (in tasks with continuous reports) most clearly in those studies that included higher memory demands, such as with four memory items (van Ede et al., 2019) or when multiple sources competed for the internal focus of attention (van Ede et al., 2020).

### Considerations on task difficulty and cognitive load

While our findings suggest a gaze bias specific to the working memory task, it is important to consider that differences in task difficulty between the control (perceptual judgment) and primary (working memory) tasks may have also contributed to the observed differences in oculomotor behavior. Although our tasks were designed to be as similar as possible in terms of bottom-up sensory input and overall task engagement, it is notoriously hard to fully equate difficulty and engagement (including things like participant motivation). In our experiment, the perception and working memory tasks relied on different performance metrics—accuracy in working memory and hit rates in perceptual judgment—making direct comparisons difficult. Yet, even if we had used the same performance measure and would have perfectly equated task performance (e.g., percentage correct), there is no guarantee that participants would have put in the same amount of effort. Indeed, reaching the same performance in the working memory task as in the perceptual task would require additional processes, such as the retention/rehearsal of the information (and because information may decay, this would require better encoding to yield the same eventual performance). Nevertheless, in future studies, it would be interesting to include even more tightly matched control conditions that better equate difficulty using, for example, an adaptive staircase procedure. At the same time, it is worth noting how our working memory condition did not merely have a stronger microsaccade bias, but how this bias was particularly prolonged in the working memory case (indeed, in the perception trials, we also noted a robust microsaccade bias immediately at encoding, without being followed by a particularly robust sustained microsaccades bias in the period that followed). The prolonged temporal profile we observed in the working memory task is at least consistent with the prolonged rehearsal demands in the working memory task.

### What opportunities and challenges do these findings bring?

Our findings demonstrate how microsaccades can be used to track working memory rehearsal through spatial location over time. This may open new avenues for characterizing the spatial–temporal trajectories of working memory rehearsal in future studies with complementary manipulations of spatial and/or temporal working memory demands. We have previously demonstrated that microsaccades are a valuable tool for studying attentional selection and prioritization of objects in visual working memory (van Ede et al., 2019; Liu et al., 2022; de Vries et al., 2023). Here, we extend this involvement to the maintenance period. Zooming out, these findings add to a growing body of work showing the utility of using microsaccades to track cognition at large, including for tracking complementary cognitive functions such as temporal expectations (Olmos-Solis et al., 2017; Amit et al., 2019; Denison et al., 2019; Tal-Perry and Yuval-Greenberg, 2021), perceptual learning (Hung and Carrasco, 2023), and decision-making (Loughnane et al., 2018).

At the same time, our data bring a practical implication to the fore that presents a challenge, rather than an opportunity. They show how microsaccades cannot be neglected in neuroscience studies of visual working memory (Liu et al., 2022, 2023b). Several recent reports already made clear how microsaccades may confound neural decoding of memory representations, by showing how microsaccades can be systematically biased by task-relevant object features such as shape and orientation (Dotson et al., 2018; Mostert et al., 2018; Quax et al., 2019; Thielen et al., 2019; Linde-Domingo and Spitzer, 2023). Building on this work, our data make clear how systematic biases in microsaccades can be a concern also for arbitrarily chosen spatial stimulus configurations at encoding, even if stimulus configurations are never explicitly relevant for the participant.

## Conclusion

In conclusion, by studying microsaccades, we provide unique evidence that the brain's oculomotor system participates in rehearsing visual objects in working memory through their incidental locations. The microsaccade-maintenance bias we observed may signal an attentional refreshing mechanism by the oculomotor system that supports visual object maintenance. At the same time, the observed decay in the observed microsaccade-maintenance bias raises intriguing questions about the dynamic nature of working memory rehearsal across time.

## Synthesis

Reviewing Editor: Ifat Levy, Yale School of Medicine Decisions are customarily a result of the Reviewing Editor and the peer reviewers coming together and discussing their recommendations until a consensus is reached. When revisions are invited, a fact-based synthesis statement explaining their decision and outlining what is needed to prepare a revision will be listed below. The following reviewer(s) agreed to reveal their identity: Ziad Hafed, Sven Ohl. Note: If this manuscript was transferred from JNeurosci and a decision was made to accept the manuscript without peer review, a brief statement to this effect will instead be what is listed below.

The manuscript presents an eye tracking study that asked how the oculomotor system relates to rehearsal in visual working memory. To this end, the authors followed up on a paradigm that has been used over the past years to study spatial gaze biases during memory maintenance after rendering the location of the encoded stimuli irrelevant for the memory task. The main new contribution of this particular study is to assess the gaze biases in a time window following encoding, hence before presentation of the post cue. The authors used a simple but very clever design to assess microsaccade rates as a function of whether they were aligned or orthogonal to the axis of two encoded stimuli. Indeed, the authors observed more microsaccades aligned with the axis where the relevant stimuli were initially encoded. Based on this main result the authors claim that microsaccades track rehearsal in visual working memory. The present study provides some additional and interesting control conditions to determine whether gaze biases following the encoding reflect a simple response to the visual transient during encoding or whether the biases are indeed involved in rehearsal. The authors observed the gaze bias only when participants were tasked to remember the stimuli (as compared to just perceiving the stimuli). This result rules out alternative explanations (e.g., a simple response to the visual transient) and supports their claim of a tight relationship between eye movements and rehearsal.

This is an interesting and well written paper, following up on an increasing number of recent studies that addressed gaze biases in visual working memory. The results are convincing and well visualized. The present findings can be nicely integrated into the general picture emerging from the studies that assessed gaze biases in visual working memory. The present manuscript complements previous findings by looking at a different time window in that basic memory task. Overall, reviewers thought that the manuscript was insightful, but had several comments and concerns that should be addressed.

- It is not clear how aligned and misaligned microsaccades were classified. Reviewers could not find any description of the procedure used - this must be explicitly stated and justified, and may be critical. Importantly, in the control conditions, the distributions of microsaccade amplitudes and directions were slightly altered compared to the memory condition. Depending on how aligned and misaligned microsaccades are defined, this could arbitrarily cause the effect to appear weaker in the control conditions.

- The authors argue for a strong link between rehearsal and microsaccades, but do not show any behavioral outcomes. Microsaccades are a very powerful tool, because they provide a time-resolved window into cognition in the absence of participants' awareness. However, to argue for a link to behavior, the authors should test for a performance benefit of the aligned vs. misaligned directions. Behavior can be measured by performance accuracy, as well as reaction time.

- Reviewers appreciated the control conditions which are critical to support the authors' conclusions. However, a mediating factor that seems very relevant is overall cognitive load, or task engagement, and it is not clear how the authors controlled for things like task difficulty. The lack of a strong gaze bias in the perception task could simply result from a too easy task, which is consistent with the higher overall accuracy in the perceptual task compared to the memory task. The authors should either add an additional control where task difficulty is controlled by a staircase procedure, or tone down their conclusions and discuss the potential role of task difficulty.

- Why did the authors restrict their analysis to a time window before the onset of the report cue? It seems important to report microsaccades rates from the entire trial. Analyzing the post cue window would allow the authors to replicate previous reports of gaze biases during selection in visual working memory. The authors could determine, for instance, whether the gaze biases are comparable in size to the ones reported in the original studies. An exploratory analyses could even link how gaze biases before the cue (during rehearsal) are associated with gaze biases after the cue.

- Related to the last point: it will be helpful to have a summary in the introduction of the authors' prior work on microsaccades and memory, and how this study is different. The authors occasionally reiterate their fundamental narrative, which the community has already embraced. The basic idea of their paradigm using gaze biases in a task where location is not relevant is powerful; however, this reasoning motivated a lot of experiments with the same paradigm over the past couple of years. It is important to shift the focus towards the novel contributions introduced by the study.

- The authors may consider additional analysis that exploits the "time-resolved" measure offered by the microsaccades. Specifically, it is possible that in this task stimulus onset introduces oscillations in microsaccade directions as a function of time. The authors can separate trials based on the direction of the first microsaccade (after, 100 ms after stimulus onset) and plot the distributions of microsaccade directions as a function of time for each direction of first microsaccade (similar to Fig. 10 of Tian et al., 2016 or Fig. 11 of Tian et al., 2018). This may reveal evidence of oscillation in microsaccade directions along the axis of the remembered targets, which could explain the somewhat mild oscillation in the rate plots. It would also justify the use of microsaccades as a "time-resolved" tool, and help generate new ideas about the potential role of the oculomotor in working memory rehearsal.

- The authors should consider the primary role of eye movements in fixation, which is to optimize eye position on the fixation spot. The oculomotor system is engaged in a dual task of both influencing cognition and controlling of eye position - this should be discussed.

Minor comments:

- Line 30: Hafed and Clark 2002 could also be cited here.

- Line 124: these couple of sentences were difficult to understand. It will also be helpful to have the rationale for the different controls already explicitly explained here.

- Line 152: this paragraph is a bit hard to follow and would benefit from plots of example saccades and their velocity traces/thresholds etc.
